# *S*LCO1B1 Variants and Angiotensin Converting Enzyme Inhibitor (Enalapril) -Induced Cough: a Pharmacogenetic Study

**DOI:** 10.1038/srep17253

**Published:** 2015-11-26

**Authors:** Jian-Quan Luo, Fa-Zhong He, Zhen-Min Wang, Ning-Ling Sun, Lu-Yan Wang, Gen-Fu Tang, Mou-Ze Liu, Qing Li, Xiao-Ping Chen, Zhao-Qian Liu, Hong-Hao Zhou, Wei Zhang

**Affiliations:** 1 Department of Clinical Pharmacology, Xiangya Hospital, Central South University, Changsha 410008; P. R. China; 2 Institute of Clinical Pharmacology, Central South University; Hunan Key Laboratory of Pharmacogenetics, Changsha 410078; P. R. China; 3 Hunan Province Cooperation Innovation Center for Molecular Target New Drug Study, Hengyang 421001, P. R. China; 4 Department of Cardiology, People’s Hospital, Peking University, Beijing 100044, P. R. China; 5 School of Health Administration, Anhui Medical University, Hefei 230032, PR China

## Abstract

Clinical observations suggest that incidence of cough in Chinese taking angiotensin converting enzyme inhibitors is much higher than other racial groups. Cough is the most common adverse reaction of enalapril. We investigate whether *SLCO1B1* genetic polymorphisms, previously reported to be important determinants of inter-individual variability in enalapril pharmacokinetics, are associated with the enalapril-induced cough. A cohort of 450 patients with essential hypertension taking 10 mg enalapril maleate were genotyped for the functional *SLCO1B1* variants, 388A > G (Asn130Asp, rs2306283) and 521T > C (Val174Ala, rs4149056). The primary endpoint was cough, which was recorded when participants were bothered by cough and respiratory symptoms during enalapril treatment without an identifiable cause. *SLCO1B1* 521C allele conferred a 2-fold relative risk of enalapril-induced cough (95% confidence interval [CI] = 1.34–3.04, *P* = 6.2 × 10^−4^), and haplotype analysis suggested the relative risk of cough was 6.94-fold (95% CI = 1.30–37.07, *P* = 0.020) in SLCO1B1*15/*15 carriers. Furthermore, there was strong evidence for a gene-dose effect (percent with cough in those with 0, 1, or 2 copy of the 521C allele: 28.2%, 42.5%, and 71.4%, trend *P* = 6.6 × 10^−4^). Our study highlights, for the first time, *SLCO1B1* variants are strongly associated with an increased risk of enalapril-induced cough. The findings will be useful to provide pharmacogenetic markers for enalapril treatment.

Hypertension is the most common disease seen in primary care and leads to stroke, myocardial infarction, renal failure, and even death if not treated appropriately^1^. The control of hypertension can be improved by increasing awareness and improving treatment of hypertension^2^. Enalapril is an angiotensin-converting enzyme (ACE) inhibitor used in the treatment of primary hypertension, heart failure, left ventricular dysfunction, and chronic kidney failure. Pharmacogenetics, as an important component of personalised or precision medicine, investigates the genetic variants determining drug response to improve drug efficacy and prevent adverse drug reactions^3^,^4^. Numerous common genetic polymorphisms on the efficiency and safety of hypertension treatment have been identified by the pharmacogenetic or pharmacogenomic approach^5^,^6^,^7^,^8^,^9^,^10^.

Common adverse reactions of ACE inhibitors include cough, increased serum creatinine, headache, dizziness, skin rash *et al.* Cough is the most common side effect of ACE-inhibitors and may occur within hours following the first dose of the medication^11^,^12^. The reported incidence of cough in patients prescribed with ACE inhibitors ranges from 5% (West) to as high as 50% or more (Chinese). A number of factors contributing to the different incidence of cough include sample size, duration of follow-up, cohort of patients enrolled, different ACE inhibitors^13^,^14^,^15^. Racial differences affect the occurrence of ACE inhibitors-induced cough. A higher incidence of cough has been reported in Chinese, compared to Caucasians^16^,^17^. To date, a variety of studies have investigated the association of candidate genetic polymorphisms with ACE inhibitors-induced cough, but no genes were confirmed to strongly predispose to ACE inhibitors-induced cough^18^,^19^,^20^,^21^. The genetic basis of ACE inhibitors-induced cough remains to be determined.

The solute carrier organic anion transporter family member 1B1 (*SLCO1B1*) gene encodes a sodium-independent bile acid transporter, the organic anion transporter protein (OATP1B1). OATP1B1 is specifically expressed at the basolateral membrane of hepatocytes and involves in hepatic clearance of many drugs, including the statins^22^,^23^,^24^, antidiabetic agents (repaglinide and nateglinide)^25^,^26^, angiotensin II receptor antagonists (valsartan and olmesartan)^27^,^28^, and ACE inhibitors (enalapril and temocapril)^29^. A total of 190 common single-nucleotide polymorphisms (SNPs) with minor allele frequency greater than 5% have been identified in the *SLCO1B1* gene^30^. Among these, two commonly occurring non-synonymous SNPs (521T > C, Val174Ala, rs4149056 and 388A > G, Asn130Asp, rs2306283) have been showed to cause an alteration in the pharmacokinetics (PK) and pharmacodynamics (PD) of the OATP1B1 substrates in our previous studies^23^,^25^. Furthermore, the *SLCO1B1* genetic variants were reported to be an important determinant of the PK of enalapril in the Chinese men population in a recent study^31^. However, there are no studies focused on the association between *SLCO1B1* functional variants and the ACE inhibitors-induced cough.

Therefore, in the present study, we set out to investigate whether the two common *SLCO1B1* genetic variants (521T > C and 388A > G) previously reported to have vital effects on the function of transporting activity are pharmacogenetic determinants of the occurrence of cough in essential hypertensive patients treated with enalapril in China.

## Results

### Descriptive characteristics and clinical features of the study population

A total of 450 subjects received the ACE inhibitor enalapril. Enalapril-induced cough occurred in one hundred and forty-four patients and these subjects were defined as coughers (144), while the others without enalapril-induced cough were classified as controls (306). The demographic and clinical characteristics of the entire cohort and those with and without the enalapril-induced cough are summarized in Table 1. Of these characteristics, sex and smoking status were significantly different between groups with or without the enalapril-induced cough, with a greater percentage of female subjects (*P* = 0.006), but a lower percentage of smoking subjects in the coughers (*P* = 0.038). No significant difference between the two groups was found for other demographic and clinical characteristics.

### Association of the *SLCO1B1* 388A > G and 521T > C variants with the risk of enalapril-induced cough

Genotype distributions of the *SLCO1B1* 388A > G and 521T > C polymorphisms among the coughers and controls are shown in Table 2. The two variants were successfully genotyped in 98.2% (388A > G) and 98.9% (521T > C) of the participants. The two *SLCO1B1* SNPs were both conformed to the Hardy-Weinberg equilibrium (*P* = 0.096 for 388A > G and *P* = 0.842 for 521T > C).

We found that the allele distribution of the 521T > C variant between the coughers and controls was statistically different (17.6% vs. 9.6%, *P* = 6.2 × 10^−4^). The combined TC/CC genotypes frequency were significantly higher among coughers than controls (31.7% vs. 18.5%, *P* = 0.002). In addition, logistic regression was conducted to evaluate the associations between *SLCO1B1* genotypes and risk of enalapril-induced cough. As shown in Table 2, compared with the TT genotype, the TC genotype had a markedly increased risk of enalapril-induced cough (adjusted OR = 1.92, 95% confidence interval (CI) = 1.19–3.09, *P* = 0.007), and the 521CC genotype conferred a nearly 6-fold risk for enalapril-induced cough (crude OR = 6.37, 95%CI = 1.22–33.37; adjusted OR = 5.67, 95%CI = 1.07–30.16). In contrast, we didn’t find any significant association between 388A > G polymorphism and the risk of enalapril-induced cough (*P* > 0.05). When the 388A > G AA homozygotes was used as the reference, the adjusted OR (95% CI) for AG, GG, and G allele carriers (AG/GG) were 0.67 (0.32–1.42), 0.65 (0.32–1.34), and 0.66 (0.33–1.34), respectively. As a significant effect of sex on the risk of enalapril-induced cough was observed, we further analyzed the association of *SLCO1B1* genotypes separately by sex (Table 3). We found that the association between *SLCO1B1* 388A > G genotypes and enalapril-induced cough remained not significant both in men and women. For the *SLCO1B1* 521T > C genotypes, in male patients, the risk of enalapril-induced cough in *SLCO1B1* 521C allele carriers was higher compared with TT genotype carriers, with marginal significance (OR = 2.06, 95%CI = 0.92–4.62*, P* = 0.065), while female patients were still strongly associated with *SLCO1B1* 521T > C genotypes distribution (OR = 2.04, 95%CI = 1.16–3.60, *P* = 0.012).

**Figure 1:**
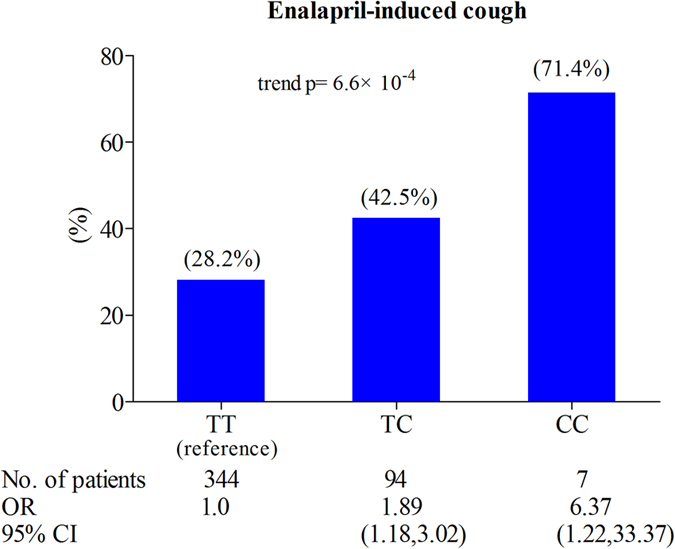
Gene-dose effects (*SLCO1B1* 521T > C) on the enalapril-induced cough. On X-axis the three *SLCO1B1* 521T > C genotypes (TT, TC, CC) are presented. On Y-axis the incidence of enalapril-induced cough is presented as percentages (%). CI, confidence interval; OR, odds ratio

A previous study indicated that there was a gene-dose effect on the association between *SLCO1B1* 521T > C allele and statin-induced side effects^32^. Thus, we sought to find a similar association of *SLCO1B1* 521T > C with the enalapril-induced cough. In the current study, strong evidence was indeed found for a gene-dose effect: the percentage of coughers increased with the increasing numbers of *SLCO1B1* 521T > C risk allele. For the *SLCO1B1* 521C allele non-carriers (n = 344), carriers of one 521C allele (n = 94), and carriers of two 521C alleles (n = 7) the proportions with the cough were 28.2%, 42.5%, and 71.4% respectively (trend *P* = 6.6 × 10^−4^) (Fig. 1).

### Linkage disequilibrium and haplotype analysis for the two *SLCO1B1* genetic polymorphisms

The linkage disequilibrium analysis showed that the two SNPs were in strong linkage disequilibrium, with a D-prime of 0.999 (r^2^ = 0.049) for *SLCO1B1* 388 A > G and 521 T > C, in our population.

Three haplotypes, that is SLCO1B1*1a (388A and 521T), SLCO1B1*1b (388G and T), SLCO1B1*15 (388G and 521C), were observed in our study. The SLCO1B1*5 (388A and 521C) haplotype was not observed, which meaned 388A and 521C allele were in a perfect linkage disequilibrium. The frequencies of the three haplotypes (*1b, *1a and *15) in the current population were 61.4, 26.3, and 12.3%, respectively. The haplotype distributions between the enalapril-induced cough and control groups are shown in Table 4. The overall haplotype frequency was statistically different between the coughers and controls (global *P* = 0.001). The frequencies of *1a, *1b, and *15 were 27.8, 54.6, and 17.6% in coughers, and 25.5, 64.7, and 9.8% in controls. The common risk haplotype, *15 (388G and 521C), was markedly higher in the coughers group (17.6%) than in the control group (9.8%) (p < 0.001), while the haplotype *1b (388G and 521T) frequency was significantly lower in the coughers (54.6%) than that in the controls (64.7%) group (*P* = 0.004). Haplotype pairs analysis suggested the risk of enalapril-induced cough was significantly higher in the patients carrying the *1b/*15, *1a/*15 or *15/*15 haplotype pairs than the patients with the reference genotype *1b/*1b (OR = 1.94, 95%CI = 1.08–3.51, *P* = 0.026; OR = 2.38, 95%CI = 1.03–5.53, *P* = 0.040; OR = 6.94, 95%CI = 1.30–37.07, *P* = 0.020, respectively). Haplotype groups analysis indicated the subjects harbouring the *15 haplotype (*1b/*15, *1a/*15, *15/*15) had a significantly increased risk for enalapril-induced cough, compared to other haplotypes (*1a/*1b, *1a/*1a, *1b/*1b) (OR = 1.99, 95%CI = 1.26–3.14, *P* = 0.003). The effect of sex on the relationship between the *SLCO1B1* haplotypes and enalapril-induced cough was further determined (Table 4). In female subjects, the common risk haplotype, *15 (388G and 521C), was significantly higher in the coughers group (18.0%) than in the control group (9.5%) (*P* = 0.004) the haplotype *1b (388G and 521T) frequency was significantly lower in the coughers (54.9%) than that in the controls (65.2%) group (*P* = 0.015), while there is no marked difference in male patients. Additionally, the female subjects with the *15 haplotype (*1b/*15, *1a/*15, *15/*15) had a significantly increased risk for enalapril-induced cough, compared to other haplotypes (*1a/*1b, *1a/*1a, *1b/*1b) (OR = 2.00, 95%CI = 1.14–3.53, *P* = 0.016), while the findings in male patients were marginally significant (OR = 2.04, 95%CI = 0.92–4.56, *P* = 0.078).

## Discussion

To the best of our knowledge, this is the first study to discuss the involvement of transport protein OATP1B1 in the occurrence of ACE inhibitors-induced cough. In this large pharmacogenetics study, we provide compelling evidence that the *SLCO1B1* functional genetic variant (521T > C; Val174Ala) substantially alters the risk of enalapril-induced cough in the Chinese population from the perspective of enalapril PK. The results indicate that the risk of enalapril-induced cough in CT heterozygous and CC homozygous carriers are nearly 2-fold and 6-fold higher compared to the TT wild type carriers. Furthermore, there is strong evidence for a gene-dose effect with the association between 521T > C polymorphism and cough. However, the 388A > G SNP is not significantly associated with enalapril-induced cough. The haplotype analysis results suggest that *SLCO1B1* *15 (388G and 521C) haplotype may be a risk factor for enalapril-induced cough.

The *SLCO1B1* 521T > C polymorphism appeared consistently functional, but results regarding the *in-vitro* functional effects of the 388A > G variant, which may make the transport activity increase, decrease, or not change, were conflicting^33^. In China, the 521T > C variant was reported to be an important determinant of the PK of enalapril, whereas no significant effect on the PK of enalapril was found for the 388A > G polymorphism^31^. Consistent with the pharmacokinetic effects, our finding showed only the *SLCO1B1* 521T > C variant was significantly associated with enalapril-induced cough.

Based on the two SNPs, three haplotypes, SLCO1B1*1a, SLCO1B1*1b and SLCO1B1*15 are constructed in our study. The functional effects of SLCO1B1*15 haplotype are well documented, and are associated with reduced hepatic uptake and increased plasma concentrations of its substrates, such as pitavastatin, irinotecan *et al.*^34^,^35^ In Chinese participants, there was significant difference in plasma concentration of enalapril between *15 carriers and *15 non-carriers after single or multiple oral doses of enalapril (10 mg daily). Most importantly, the accumulation ratio of enalaprilat in *15 carriers was also markedly higher than that in *15 non-carriers treated with enalapril 10 mg/d for 7 days^31^. In accordance with these results, our findings of haplotype analysis showed the risk of enalapril-induced cough to be 2-fold higher in patients carrying the *1b/*15 or *1a/*15 haplotype pairs and the risk of enalapril-induced cough to be nearly 7-fold higher in patients carrying the *15/*15 haplotype pairs than the patients with the reference haplotype *1b/*1b. The frequency of *SLCO1B1* haplotypes seems to be dependent on the ethnic population^30^,^36^,^37^. The frequency of the SLCO1B1*15 haplotype was 12.3% in our current study, similar to that in Asians, but greater than that of Caucasians (2.4%)^36^. Therefore, the different frequencies of *SLCO1B1* haplotypes may explain the different incidence of enalapril-induced cough in different racial populations.

ACE inhibitors-induced cough was reported decades ago, but the exact mechanisms for the side effect are still unknown. Several clinical risk characteristics for ACE inhibitors-induced cough were identified such as female sex, Chinese origin, and smoking habits^12^,^16^,^17^,^21^,^38^. Our findings of clinical characteristics are in perfect agreement with the well-established impact of female sex and smoking on the risk of ACE inhibitors-induced cough. This influence may result from a more sensitive cough reflex sensitivity in females and nonsmokers^39^,^40^,^41^, but the precise mechanism remains to be elucidated in future studies. Previous data clearly suggest that ACE inhibitors-induced cough is a class effect, but the occurrence of cough among the ACE inhibitors is significantly different. The incidence of enalapri-induced cough is relatively higher than that of imidapril-induced or perindopril-induced cough^13^,^14^. One of the strengths in the current pharmacogenetic study is that we focus on the enalapril-induced cough and it may eliminate the heterogeneity of different ACE inhibitors-induced cough.

In previous reports, the *SLCO1B1* genetic variants were proved to be relevant to the response to drugs transported by OATP1B1, such as statin-induced myopathy, irinotecan-induced toxicities, methotrexate clearance^42^,^43^,^44^,^45^,^46^,^47^,^48^. The clinical significance of *SLCO1B1* pharmacogenetics was best exemplified by its impact on simvastatin therapy. The FDA recommends patients carrying the C allele at *SLCO1B1* 521T > C, are recommended for a lower dose (40mg daily) because the myopathy risk is increased in these individuals. If optimal efficacy with a lower dose is not achieved, an alternate agent should be considered. Our pharmacogenomics study regarding the association between *SLCO1B1* genetic polymorphisms and ACE inhibitor enalapril-induced cough may add a novel potential for implementation of *SLCO1B1* pharmacogenetics into clinical practice.

There are several limitations to our study. Firstly, the novel findings need to be replicated in external populations, taking into account that the incidence of cough and the frequency of variations in *SLCO1B1* differ among ethnic groups. Also, further research is necessary to determine whether the different frequencies of *SLCO1B1* variants account for the prevalence of cough in disparate populations. Secondly, these results are not likely to apply to other ACE inhibitors because the substrates (ACE inhibitors) of OATP1B1 are restricted to enalapril and temocapril^27^,^29^,^31^. Further studies for the association between *SLCO1B1* and other ACE inhibitors are needed. Thirdly, the study duration of follow-up was only two weeks. Nevertheless, in current study, the occurrence of enalapril-induced cough (32%) is in accordance with an earlier report (37.8%) of a prospective study with a follow-up length of eight weeks in Han Chinese patients with hypertension^49^. Last but not least, plasma concentrations of enalapril were not measured. An in-depth analysis of the relationships between SLCO1B1 variants and plasma concentrations of enalapril in patients is needed. The current findings would be strengthened by inclusion of enalapril concentrations even if OATP1B1 functional status affected enalapril pharmacokinetics in a previous study^30^.

In summary, our findings propose that common genetic variants in *SLCO1B1* may serve as a novel pharmacogenetic biomarker for enalapril-induced cough in Chinese essential hypertensive patients because the *SLCO1B1* 521T > C polymorphism was associated with an increased risk of enalapril-induced cough. In the future, if confirmed, genotyping of SLCO1B1 variants may be useful to achieve the benefits of enalapril treatment more effectively and safely. The association between *SLCO1B1* genetic polymorphisms and enalapril-induced cough may result from the PK mechanism, which ultimately control the plasma levels of enalapril.

## Methods

### Subjects

The study protocol was conducted in accordance with the Declaration of Helsinki Principles. Ethics approval was granted for the study protocol from the Ethical Committee of the Institute of Clinical Pharmacology, Central South University. The registration number (ChiCTR-OCH-12002611) and the trial protocol were validated in the Chinese Clinical Trial Register. Written informed consent was obtained from each participating patient. Inclusion criteria were as follows: subjects of Han Chinese origin, male or female, a systolic blood pressure (SBP) of 140 mm Hg or more, and/or a diastolic blood pressure (DBP) of 90 mm Hg or more. Eligible subjects prescribed with enalapril were interviewed by the same interviewer after a 2-week period therapy. The basic clinical information of the participants, such as gender, age, waist/hip ratio(WHR), body mass index(BMI), SBP, DBP, smoking status, ethanol intake, and routine clinical laboratory results *et al.* has been recorded. Side effects including cough, dizziness, skin rash, muscle cramps, non-specific gastrointestinal symptom, elevated creatinine, and angioedema, were also collected. The participants and the interviewer were not aware of which symptom was the focus, so that the interview remained unbiased. Enalapril-induced cough was recorded when the subjects were bothered by cough and respiratory symptoms during the two-week therapy with enalapril without an identifiable cause. Cough caused by other conditions (acute respiratory infection or other respiratory diseases) or medications other than enalapril was not regarded as enalapril-induced cough.

### Genotyping

Blood samples were collected from all the study subjects in a sterile tube containing EDTA, and genomic DNA was extracted from peripheral blood leukocytes. The DNA samples were stored at −80 °C until genotyping. All individuals included in the present study were genotyped for the two non-synonymous variants *SLCO1B1* c.521T > C and *SLCO1B1* c.388A > G using the matrix-assisted laser desorption/ionization time-of-flight mass spectrometry (MALDI-TOF MS) and Sequenom MassARRAY system (Sequenom, San Diego, CA, USA). Genotyping was determined without knowledge of the controls/coughers status of subjects. The accuracy of the two SNPs genotyping data was validated by PCR-products directed sequencing of 5% masked, random samples of the patients.

### Statistical analysis

Demographic and clinical variables between coughers and control groups were analyzed using the SPSS v.19.0 (SPSS Inc., Chicago, IL, USA). The Student’s t-test when variables had a normal distribution or Mann–Whitney U-test when variables deviated from normal distribution was used to assess the numeric variables, whereas the chi-square test was used for categorical variables between the two groups. The genotype distribution of polymorphisms was tested for deviation from Hardy–Weinberg equilibrium using the chi-square goodness-of-fit test with one degree of freedom. The pairwise linkage disequilibrium (LD) estimation and haplotype reconstruction were carried out on SHEsis (http://analysis2.bio-x.cn/myAnalysis.php)^50^,^51^. The chi-square test was also used to compare the discrepancies of allele and genotype frequency between coughers and controls. The association of *SLCO1B1* polymorphisms with enalapril-induced cough was further analyzed using an unconditional logistic regression model. The odds ratios (ORs) and their 95% confidence intervals (CIs) were calculated. A 2-sided p-value less than 0.05 was considered statistically significant.

## Additional Information

**How to cite this article**: Luo, J.-Q. *et al.* SLCO1B1 Variants and Angiotensin Converting Enzyme Inhibitor (Enalapril) -Induced Cough: a Pharmacogenetic Study. *Sci. Rep.* **5**, 17253; doi: 10.1038/srep17253 (2015).

## Figures and Tables

**Table 1:** Descriptive characteristics of the study population.

Characteristics	All (n = 450)	Coughers (n = 144)	Controls (n = 306)
Sex			
Male, n (%)	166 (36.9)	40 (27.8)	126 (41.2)
Female, n (%)	284 (63.1)	104 (72.2)	180 (58.8)
Age (years)	60.2 ± 6.1	60.4 ± 5.8	60.1 ± 6.2
WHR	0.88 ± 0.07	0.89 ± 0.07	0.88 ± 0.07
BMI (Kg/m^2^)	24.79 ± 3.46	23.99 ± 3.41	23.57 ± 4.38
FPG (mmol/L)	5.67 ± 1.88	5.65 ± 1.59	5.68 ± 2.00
TC (mmol/L)	4.82 ± 0.97	4.94 ± 0.99	4.76 ± 0.96
TG (mmol/L)	1.50 ± 1.06	1.45 ± 0.72	1.52 ± 1.19
SCr (umol/L)	67.43 ± 26.77	64.05 ± 18.52	69.02 ± 29.77
SBP (mm Hg)	165.5 ± 17.7	167.4 ± 17.0	164.5 ± 18.0
DBP (mm Hg)	92.6 ± 11.2	92.6 ± 10.9	92.6 ± 11.3
HR (beats/min)	74.5 ± 9.9	74.1 ± 9.5	74.8 ± 10.1
Smoking status			
Smokers, n (%)	129 (28.7)	32 (22.2)	97 (31.7)
Non-smokers, n (%)	321 (71.3)	112 (77.8)	209 (68.3)

Abbreviations: WHR, waist-hip ratio; BMI, body mass index; FPG, fasting plasma glucose; TC, total cholesterol; TG, triglyceride; SCr, serum creatinine; SBP, systolic blood pressure; DBP, diastolic blood pressure; HR, heart rate. Values were expressed as mean ± S.D.

**Table 2:** Association of *SLCO1B1* genetic polymorphisms with the risk of enalapril-induced cough.

	Coughers^a^ n(%)	Controls^a^ n(%)	Without Adjustment*		With Adjustment†	
			*P*-value	Crude OR(95%CI)^b^	*P*-value	Adjusted OR(95%CI)^b^
388 A > G						
AA	15(10.5)	22(7.4)		1.00(reference)		1.00(reference)
AG	49(34.3)	108(36.1)	0.278	0.67(0.32–1.39)	0.295	0.67(0.32–1.42)
GG	79(55.2)	169(56.5)	0.295	0.69(0.34–1.39)	0.246	0.65(0.32–1.34)
AG + GG	128(89.5)	277(92.6)	0.266	0.68(0.34–1.35)	0.251	0.66(0.33–1.34)
A allele	79(27.6)	152(25.4)		1.00(reference)		
G allele	207(72.4)	446(74.6)	0.485	0.89 (0.65,1.23)	NC	NC
521 T > C						
TT	97(68.3)	247(81.5)		1.00(reference)		1.00(reference)
TC	40(28.2)	54(17.8)	0.008	1.89 (1.18,3.02)	0.007	1.92 (1.19,3.09)
CC	5(3.5)	2(0.7)	0.024	6.37(1.22,33.37)	0.042	5.67 (1.07,30.16)
TC + CC	45(31.7)	56(18.5)	0.002	2.05(1.30,3.23)	0.002	2.07 (1.30,3.29)
T allele	234(82.4)	548(90.4)		1.00(reference)		
C allele	50(17.6)	58(9.6)	6.2 × 10^−4^	2.02(1.34,3.04)	NC	NC

Homozygous wild-type patients served as the reference group. NC, not calculated.

^a^Number of subjects (percent).

^b^OR = odds ratio; CI = confidence interval.

^*^Uncorrected *P* value and crude OR using χ^2^ tests with Pearson 2 × 2 test or Fisher exact test.

^†^Adjusted data by multivariate logistic regression analysis for sex, and smoking status.

**Table 3:** The effect of sex on the association between *SLCO1B1* genetic polymorphisms and enalapril-induced cough.

	Male		*P*-value	OR(95%CI)^b^	Female		*P*-value	OR(95%CI)^b^
	Coughers^a^ n(%)	Controls^a^ n(%)			Coughers^a^ n(%)	Controls^a^ n(%)		
388 A > G								
AA	5(12.8)	9(7.3)		1.00(reference)	10(9.6)	13(7.4)		1.00(reference)
AG	13(33.3)	46(37.4)	0.312	0.50(0.14,1.78)	36(34.6)	62(35.2)	0.549	0.81(0.32,2.03)
GG	21(53.8)	68(55.3)	0.336	0.52(0.15,1.75)	58(55.8)	101(57.4)	0.517	0.74(0.30,1.79)
AG + GG	34(87. 2)	114(92.7)	0.328	0.52(0.16,1.67)	94(90.4)	163(92.6)	0.512	0.76(0.32,1.81)
A allele	23(29.5)	64(26.0)		1.00(reference)	56(0.269)	88(0.250)		1.00(reference)
G allele	55(70.5)	182(74.0)	0.547	1.19(0.68,2.09)	152(0.731)	264(0.750)	0.615	1.11(0.75,1.63)
521 T > C								
TT	26(66.7)	101(80.8)		1.00(reference)	71(68.9)	146(82.0)		1.00(reference)
TC	13(33.3)	23(18.4)	0.052	2.15(0.95,4.84)	27(26.2)	31(17.4)	0.051	1.78(0.99,3.21)
CC	0(0.0)	1(0.8)	NC	NC	5(4.9)	1(0.6)	0.018	10.0(1.15,87.24)
TC + CC	13(33.3)	24(19.2)	0.065	2.06(0.92,4.62)	32(31.1)	32(18.0)	0.012	2.04(1.16,3.60)
T allele	65(83.3)	225(90.0)		1.00(reference)	169(82.0)	323(90.7)		1.00(reference)
C allele	13(16.7)	25(10.0)	0.108	1.80(0.87,3.72)	37(18.0)	33(9.3)	0.003	2.14(1.29,3.55)

^a^Number of subjects (percent).

^b^OR = odds ratio; CI = confidence interval; Data are calculated by multivariate logistic regression analysis for smoking status.

**Table 4:** Haplotype distributions of *SLCO1B1* genetic polymorphisms.

		Total		*P*	OR(95%CI)^c^	Male		*P*	OR(95%CI)^c^	Female		*P*	OR(95%CI)^c^
Haplotype analysis^a^		Coughers^b^	Controls^b^			Coughers^b^	Controls^b^			Coughers^b^	Controls^b^		
Haplotype^d^	*1a	79(27.8)	151(25.5)	0.466	1.13(0.82,1.55)	23 (29.5)	63(25.8)	0.524	1.20(0.68,2.11)	56(27.2)	88(25.3)	0.622	1.10(0.75,1.63)
	*1b	155(54.6)	383(64.7)	0.004	0.66(0.49,0.87)	42 (53.9)	156(63.9)	0.111	0.66(0.39,1.10)	113(54.9)	227(65.2)	0.015	0.65(0.46,0.92)
	*15	50(17.6)	58(9.8)	< 0.001	1.97(1.31,2.96)	13(16.7)	25(10.2)	0.126	1.75(0.85,3.62)	37(18.0)	33(9.5)	0.004	2.09(1.26,3.45)
Haplotype pairs	*1b/*1b	45(31.7)	125(42.2)		1.00(reference)	12(30.8)	50(41.0)		1.00(reference)	33(32.0)	75(43.1)		1.00(reference)
	*1a/*1b	37(26.1)	93(31.4)	0.701	1.11 (0.66,1.84)	9(23.1)	39(32.0)	0.936	0.96(0.37,2.51)	28(27.2)	54(31.0)	0.600	1.18(0.64,2.18)
	*1a/*1a	15(10.6)	22(7.4)	0.087	1.89 (0.90,3.97)	5(12.8)	9(7.4)	0.284	2.32(0.66,8.18)	10(9.7)	13(7.5)	0.231	1.75(0.70,4.39)
	*1b/*15	28(19.7)	40(13.5)	0.026	1.94 (1.08,3.51)	9(23.1)	17(13.9)	0.125	2.21(0.79,6.15)	19(18.4)	23(13.2)	0.090	1.88(0.90,3.91)
	*1a/*15	12(8.5)	14(4.7)	0.040	2.38 (1.03,5.53)	4(10.3)	6(4.9)	0.214	2.78(0.68,11.42)	8(7.8)	8(4.6)	0.123	2.27(0.79,6.57)
	*15/*15	5(3.5)	2(0.7)	0.020	6.94(1.30,37.07)	0(0.0)	1(0.8)	NC	NC	5(4.9)	1(0.6)	0.015	11.36(1.28,101.10)
Haplotype groups	-/-	97(68.3)	240(81.1)		1.00(reference)	26(66.7)	98(80.3)		1.00(reference)	71(68.9)	142(81.6)		1.00(reference)
	*15/-	45(31.7)	56(18.9)	0.003	1.99(1.26,3.14)	13(33.3)	24(19.7)	0.078	2.04(0.92,4.56)	32(31.1)	32(18.4)	0.016	2.00(1.14,3.53)

^a^*1a = 388A and 521T; *1b = 388G and 521T; *15 = 388G and 521C. -/- including (*1b/*1b,*1a/*1b, *1a/*1a). *15/- indicates at least one*15 allele (*1b/*15, *1a/*15, *15/*15).

^b^Data were expressed as number of subjects (percent).

^c^OR = odds ratio; CI = confidence interval; NC = not calculated.

^d^The *P*-value was calculated compared to other haplotypes. P value was calculated using χ^2^ tests with Pearson 2 × 2 test or Fisher exact test.
